# Plexin-B3 Regulates Cellular Motility, Invasiveness, and Metastasis in Pancreatic Cancer

**DOI:** 10.3390/cancers13040818

**Published:** 2021-02-16

**Authors:** Sugandha Saxena, Dipakkumar R. Prajapati, Paran Goel, Babita Tomar, Yuri Hayashi, Pranita Atri, Satyanarayana Rachagani, Paul M. Grandgenett, Michael A. Hollingsworth, Surinder K. Batra, Rakesh K. Singh

**Affiliations:** 1 Department of Pathology and Microbiology, Nebraska Medical Center, Omaha, NE 68198, USA; sugandha.saxena@unmc.edu (S.S.); dipakkumar.prajapati@unmc.edu (D.R.P.); paran.goel@unmc.edu (P.G.); Babita.tomar0404@gmail.com (B.T.); yutty_2@yahoo.co.jp (Y.H.); 2 Department of Biochemistry and Molecular Biology, Nebraska Medical Center, Omaha, NE 68198, USA; p.atri@unmc.edu (P.A.); srachagani@unmc.edu (S.R.); sbatra@unmc.edu (S.K.B.); 3 Eppley Institute for Research in Cancer and Allied Diseases, Fred & Pamela Buffett Cancer Center, University of Nebraska Medical Center, Omaha, NE 68198, USA; pgrandgenett@unmc.edu (P.M.G.); mahollin@unmc.edu (M.A.H.)

**Keywords:** Plexin-B3, cellular motility, pancreatic cancer, metastasis, cancer stem cells

## Abstract

**Simple Summary:**

Plexins and their ligands Semaphorins are considered versatile regulators of cancer cell migration, angiogenesis, invasion, and metastasis. Previously our group identified Semaphorin-5A (SEMA5A), the ligand of Plexin-B3, involved in organ-specific homing of pancreatic cancer (PC) cells and plays a significant role in PC angiogenesis and metastasis. In this study, we delineate its receptor Plexin-B3 function and pathological expression in PC progression and metastasis. Our data demonstrate that impairment in the Plexin-B3 axis enhances cell motility and PC cells’ invasiveness, resulting in higher metastasis. Thus, SEMA5A/Plexin-B3 represents an attractive targetable axis in PC metastasis.

**Abstract:**

The Plexins family of proteins are well-characterized transmembrane receptors of semaphorins, axon guidance cue molecules, that mediate the cell attraction or repelling effects for such cues. Plexins and their ligands are involved in numerous cellular activities, such as motility, invasion, and adhesion to the basement membrane. The detachment of cells and the gain in motility and invasion are hallmarks of the cancer metastasis cascade, thus generating interest in exploring the role of plexins in cancer metastasis. Semaphorin–plexin complexes can act as tumor promoters or suppressors, depending upon the cancer type, and are under investigation for therapeutic purposes. Our group has identified Semaphorin-5A (SEMA5A)/Plexin-B3 as an attractive targetable complex for pancreatic cancer (PC) metastasis. However, our understanding of the Plexin-B3 function and pathological expression in PC is limited, and our present study delineates the role of Plexin-B3 in PC malignancy. We examined the pathological expression of Plexin-B3 in PC tumors and metastasis using a human tissue microarray, disease progression model of PDX-Cre-Kras^(G12D)^ (KC) mice, and different metastatic sites obtained from the Kras^G12D^; Trp53^R172H^; Pdx1-Cre (KPC) mice model. We observed a higher Plexin-B3 expression in PC tumor cores than the normal pancreas, and different metastatic sites were positive for Plexin-B3 expression. However, in the KC mice model, the Plexin-B3 expression increased initially and then decreased with the disease progression. Next, to evaluate the functional role of Plexin-B3, we utilized T3M-4- and CD18/HPAF-Control and -Plexin B3 knockdown cells for different in vivo and in vitro studies. The knockdown of Plexin-B3 enhanced the in vitro cellular migration, invasiveness, and impaired colony formation in three-dimensional culture, along with an increase in cellular spread and remodeling of the actin filaments. We also observed a higher metastasis in nude mice injected with T3M-4- and CD18/HPAF-shPlexin-B3 cells compared to their respective control cells. Furthermore, we observed a lower number of proliferating Ki-67-positive cells and higher ALDH1-A1-positive cells in the tumors formed by Plexin-B3 knockdown cells compared to tumors formed by the control cells. Together, our data suggest that the loss of Plexin-B3 is associated with the interference of cell division machinery and the induction of stem cell-like characteristics in PC cells.

## 1. Introduction

Pancreatic cancer (PC) is the fourth-leading cause of cancer-related deaths, with projections to become the second-leading cause by 2030 [1]. The National Cancer Institute reports nearly equal numbers of estimated new cases and expected deaths in the United States from PC every year, statistics that have not changed in almost 50 years [2]. Most cases present themselves with either lymph nodes or liver metastases, indicating the spread of cancer to nearby and distant places by the process called metastasis [3,4]. Thus, identifying molecules that can target the metastatic cascade is highly warranted.

The gain of cancer cells’ migratory ability is critical in the metastatic cascade [5,6]. Guidance cue molecules, such as Plexins and their ligands, Semaphorins [7], regulate physiological and pathological cell migration, thus representing potential targets in the process of metastasis [8]. The recent literature reports the aberrant expression and deregulated signaling of guidance cue molecules in cancer progression [9,10,11]. Previous reports from our laboratory provide evidence for Plexin-B3 ligand–Semaphorin-5A (SEMA5A) involvement in the pathogenesis of PC metastasis [12,13,14,15]. However, a better understanding of the Plexin-B3/SEMA5A-initiated downstream signaling events and regulation of cellular phenotypes, characterizing the role of the receptor Plexin-B3 in PC, is also essential.

Initially identified as cell adhesion molecules, the Plexin family comprises four subfamilies named Plexin A, B, C, and D in vertebrates and two subfamilies named Plexin A and B in invertebrates [16]. Plexins are well-known receptors for semaphorin ligands, and both the ligand and the receptor share the characteristic “sema” domain. Plexins can either exist in membrane-bound or secreted forms known to mediate autocrine or paracrine signaling on binding to their respective semaphorins [12,16]. Plexins can also mediate an autoinhibitory effect in the ligand’s absence by their extracellular sema domain [17]. The intracellular region of Plexin molecules contains two highly conserved GTPase-activating proteins (GAP-like domains) and a linker region with no intrinsic GAP activity [16,18]. Additional to the above domains, Class B Plexins also have a C-terminal consensus sequence that interacts with PDZ domains. 

With the primary objective of analyzing the role of Plexin-B3 in PC, we analyzed the pathological expression of Plexin-B3 and characterized the functional role by utilizing different in vivo and in vitro techniques. Our results suggest that the context-dependent pathological expression of Plexin-B3 in PC and the loss of Plexin-B3 results in increased in vitro and in vivo migratory potential that can directly or indirectly result in the induction of cancer stem cell-like characteristics.

## 2. Results

### 2.1. Plexin-B3 Expression in Pancreatic Tumors Depends on the Stage of Disease Progression

**Figure 1:**
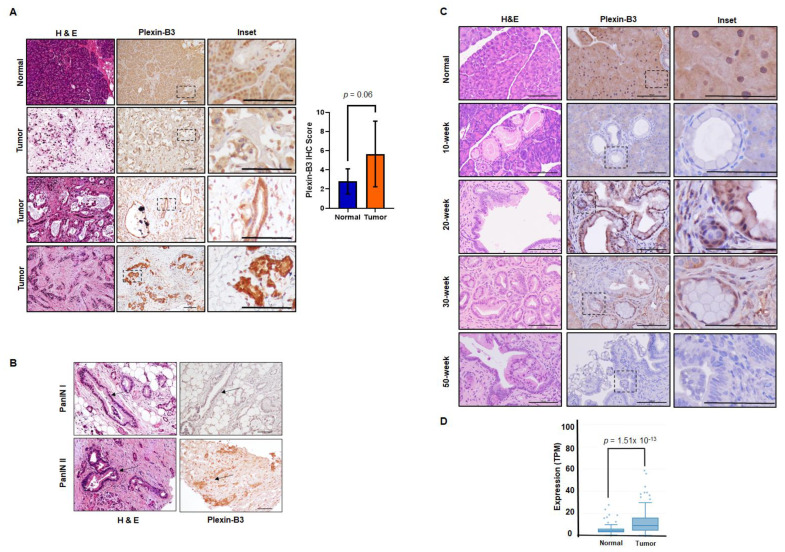
Pathological expression of Plexin-B3 in pancreatic cancer (PC). (**A**) Representative images of Plexin-B3 Immunohistochemistry (IHC), along with the corresponding Hematoxylin and eosin stain (H&E) of the normal human pancreas and tumor cores present in the tissue microarray. The pictorial graph demonstrates the different intensities of Plexin-B3 staining in pancreatic tumor tissues. The normal pancreas is positive for the expression of Plexin-B3. The scale bar represents 100 μm. Bar graph showing higher Plexin-B3 IHC scores in the tumor in comparison with a normal pancreas. (**B**) Representative images demonstrating Plexin-B3-positive PanIN I and II lesions in tumors. The scale bar represents 100 μm. (**C**) Representative images of Plexin-B3 IHC performed on a progression model derived from tumors of Pdx1-cre; LSL-Kras^(G12D)^ (KC) mice (*n* = 5) at different ages (10, 20, 30, and 50 weeks). The pictorial graph demonstrates a progressive increase in qualitative Plexin-B3 expression in cancerous lesions of the KC mice model until 20 weeks, followed by a loss of expression in the later stages of pancreatic ductal adenocarcinoma (PDAC). The normal pancreas of 50-week-old Pdx1-cre mice are positive for Plexin-B3 expression. The scale bar represents 100 μm. (**D**) Plexin-B3 gene expression analysis in the online Michigan Portal for the Analysis of NGS Data (MiPanda) showing a higher Plexin-B3 expression (*p* = 1.51 × 10^−13^) in pancreatic tumors (*n* = 419) in comparison with a normal pancreas.

We utilized a human tissue microarray consisting of a pancreatic tumor and normal pancreas cores to analyze the pathological expression of Plexin-B3 in PC. In the tumor cores, both cancer cells and tumor stroma were positive for Plexin-B3 expression (Figure 1A), and different tumors demonstrated a range of Plexin-B3 intensity (Figure 1A). Besides, we observed PanIN I and PanIN II positive for Plexin-B3 expression in the tumor cores present in the tissue microarray (Figure 1B). There was no example of PanIN III in the tumor cores of the tissue microarray. The normal human pancreas was also positive for Plexin-B3 expression. We observed a comprehensive range of Plexin-B3 Immunohistochemistry (IHC) scores in tumors compared to the normal pancreas (Figure 1A).

To gain an insight into the Plexin-B3 expression in the pancreatic ductal adenocarcinoma (PDAC) disease progression model, we analyzed pancreatic tumor tissues derived from the Kras^G12D^;Pdx1-Cre (KC) mouse model at different ages (10, 20, 30, and 50 weeks). A qualitative immunochemistry analysis of Plexin-B3 expression in the KC pancreatic tumor progression model shows an initial increase of Plexin-B3 staining until 20 weeks, followed by a decrease in Plexin-B3 expression in later stages. The normal pancreas of mice was found positive for Plexin-B3 expression (Figure 1C). We found Plexin-B3 expression localized on the membrane of tumor cells with positive staining in the surrounding tumor stroma. Apart from visualizing Plexin-B3 expression in the tumor and surrounding stroma, we also explored Plexin-B3 expression in sequenced PC tumors through the online Michigan Portal for the Analysis of NGS Data (MiPanda). We found a significantly higher Plexin-B3 expression (*p* = 1.51 × 10^−13^) in primary tumors than in a normal pancreas (Figure 1D). However, the overall survival and disease-free survival analysis with respect to Plexin-B3 expression in PC patients from the Gene Expression Profiling Interactive Analysis (GEPIA) database did not yield significant results (Figure S1A,B).

### 2.2. Different Metastatic Sites Are Positive for Plexin-B3 Expression in PC

**Figure 2:**
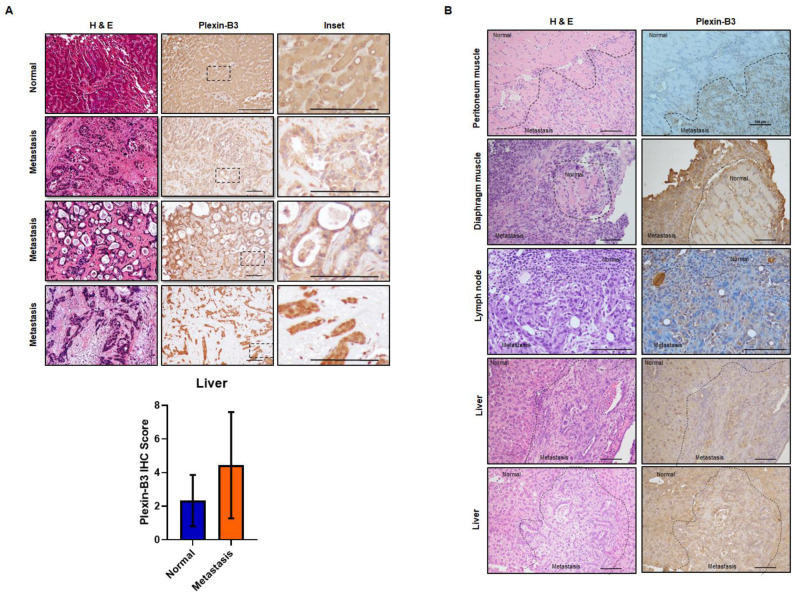
Pathological expression of Plexin-B3 in the metastatic sites of PC. (**A**) Representative images of Plexin-B3 IHC, along with the corresponding H&E of human normal liver and liver metastatic cores present in the tissue microarray. The normal liver is positive for the expression of Plexin-B3. The scale bar represents 100 μm. Bar graph showing higher Plexin-B3 IHC scores in metastatic lesions in comparison with normal liver tissue. (**B**) Representative images of Plexin-B3 IHC performed on different metastatic sites (lymph node (*n* = 1), liver (*n* = 5), diaphragm muscle (*n* = 1), and peritoneum muscle (*n* = 2)) derived from the progression model of Kras^G12D^; Trp53^R172H^; Pdx1-Cre (KPC) mice. The scale bar represents 100 μm.

To analyze the expression of Plexin-B3 at different metastatic sites in PC patients, we utilized the tissue microarray containing matched primary pancreatic tumor cores with the corresponding liver metastatic site cores. Similar to our observations of Plexin-B3 expression in primary tumors, we found a range of Plexin-B3 intensity in metastatic liver sites in PC patients (Figure 2A). Nevertheless, the range of Plexin-B3 showed a higher expression trend in metastatic liver sites in comparison with primary pancreatic tumors; however, this was not a statistically significant observation. Additionally, the correlation of Plexin-B3 expression between the matched primary pancreatic tumor cores and liver metastatic site cores was 0.56. To further evaluate the metastatic sites apart from the liver for Plexin-B3 expression, we utilized tissue sections from different metastatic sites of the Kras^G12D^; Trp53^R172H^; Pdx1-Cre (KPC) mice model. We observed a positive Plexin-B3 expression in all the evaluated metastatic sites (Figure 2B), including the liver, lymph node, peritoneum, and diaphragm, from KPC mice sacrificed at the 50-week time point.

### 2.3. Loss of Plexin-B3 Expression in PC Cell Lines Increases Cellular Spread, Motility, and Invasiveness

**Figure 3:**
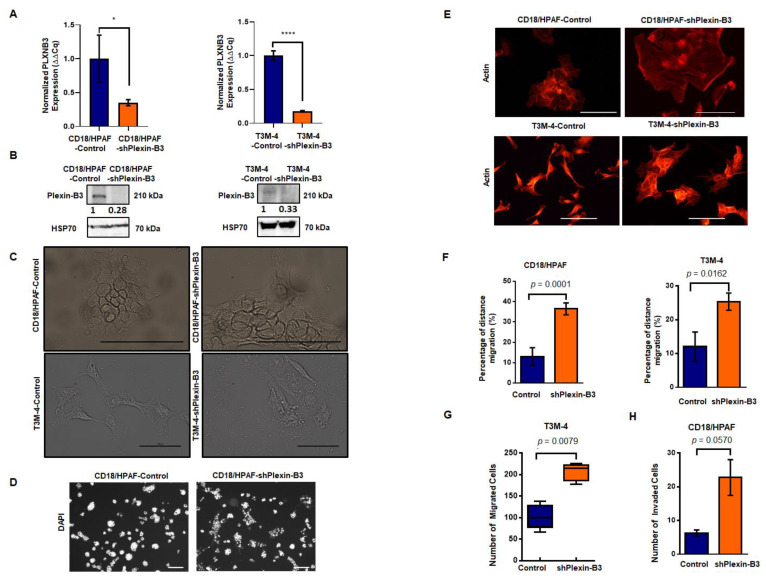
Knockdown of Plexin-B3 leads to a change in cellular morphology and increased cellular motility and invasiveness of PC cells. (**A**) Bar graph showing the relative fold decrease in mRNA expression of *PLXNB3* in CD18/HPAF- and T3M4-shPlexin-B3 samples compared with their respective controls. *HPRT* (hypoxanthine-guanine phosphoribosyltransferase) is used as a control. The values are the mean relative fold changes ± standard error of the mean (SEM, bars). (**B**) Western blot analysis of whole-cell lysates of CD18/HPAF-and T3M-4- and control and Plexin B3 knockdown cells. The Plexin-B3 expression is downregulated in Plexin-B3 knockdown cells of T3M-4- and CD18/HPAF compared to their respective control cells. (**C**) Representative images of the morphology of CD18/HPAF control and Plexin-B3 knockdown cells and T3M-4- Control and Plexin-B3 knockdown cells. Images show morphological differences with an increase in cellular area with the knockdown of Plexin-B3. The scale bar represents 100 μm. (**D**) Representative images of colonies formed by CD18/HPAF-Control and CD18/HPAF-shPlexin-B3 cells in a three-dimensional Matrigel matrix. Images show stained nuclei (DAPI) of the cells in a colony, demonstrating a lack of CD18/HPAF Plexin-B3 cell ability to form dense colonies in comparison with their respective control cells. Images are taken at 100× magnification using a Life EVOS FL fluorescence microscope. (**E**) Representative images of the actin cytoskeleton in CD18/HPAF-Control and shPlexin-B3 and T3M-4-Control and T3M-4-shPlexin-B3 cells showing remodeling of the actin cytoskeleton. The scale bar represents 100 μm in length. (**F**) Bar graphs showing a higher in vitro migration of Plexin-B3 knockdown cells in CD18/HPAF (*p* < 0.0001) and T3M-4 (*p* = 0.0162) in comparison with their respective control cells using a wound scratch assay. The values in the graph represent the percentage of distance migration, and the error bars represent the SEM. The statistical *p*-value was calculated using a Student’s *t*-test. (**G**) Box plot analysis shows a higher in vitro migration of Plexin-B3 knockdown cells in T3M-4 (*p* = 0.0078) compared to the control cells using the Transwell migration assay. The values represent the number of migrated cells, and error bars in the box plot represent the SEM. The statistical *p*-value was calculated using a nonparametric Mann–Whitney *U* Test. (**H**) Bar graph showing the number of invaded cells in the control and Plexin-B3 knockdown in CD18/HPAF cells. Bar graphs show a higher number of cells invading through the matrix in CD18/HPAF-Plexin-B3 (*p* = 0.057) knockdown cells compared to their respective control cells. The values in the graph represent the number of invaded cells, and the error bars represent the SEM. The statistical *p*-value was calculated using a nonparametric Mann–Whitney *U* Test. (* *p* ≤ 0.05, and **** *p* ≤ 0.0001).

We stably downregulated the expression of Plexin-B3 in T3M-4 and CD18/HPAF cells, as confirmed at the RNA (Figure 3A) and protein levels (Figure 3B and Figure S2). With the loss of Plexin-B3, we observed a marked difference in the morphology of T3M4-shPlexin-B3 (Figure 3C) and CD18/HPAF-shPlexin-B3 cells (Figure 3C) in comparison with their respective control cells. Plexin-B3 knockdown cells showed a flat morphology with an increase in the cellular spread area compared to their respective control cells. We also observed a lack of compact colony formation ability of CD18/HPAF-shPlexin-B3 cells grown on a three-dimensional matrix compared to their respective CD18/HPAF-Control cells (Figure 3D). However, T3M-4-Control and T3M-4-shPlexin-B3 did not show any prominent differences in compact colony formation ability when grown on a three-dimensional matrix (Figure S3). Staining the Plexin-B3 knockdown and their respective control cell lines for actin filament also showed an increased cellular spread of the cells and remodeled cytoskeleton with the loss of Plexin-B3 (Figure 3E).

Next, we evaluated the effect of Plexin-B3 knockdown on functional properties like cellular migration and invasion. In vitro cellular migration was assessed by performing a wound scratch assay and Transwell migration assay. We observed a higher in vitro cellular migration for CD18/HPAF-shPlexin-B3 (*p* < 0.0001)) (Figure 3F) and T3M-4-shPlexin-B3 (*p* = 0.0162) (Figure 3F) in comparison with their respective control cells using a wound scratch assay. We observed a similar increase in the number of T3M-4-shPlexin-B3 migrated cells than T3M-4-Control cells using a Transwell migration assay (*p* = 0.0078) (Figure 3G). We also assessed the invasion properties of these cells by using the Transwell invasion assay. We used Matrigel-coated wells for the evaluation of CD18/HPAF-shPlexin-B3 and CD18/HPAF-Control cells in this assay. We found an increase (*p* = 0.057) in the invasiveness of CD18/HPAF Plexin-B3 knockdown cells compared to CD18/HPAF-Control (Figure 3H). However, we observed no statistical difference (*p* = 0.067) in the number of invaded cells between T3M-4-Control and T3M-4-shPlexin-B3 cells (Figure S4).

### 2.4. Plexin-B3 Knockdown Inhibited Primary Pancreatic Tumor Burden in A Cell Line-Dependent Manner

**Figure 4:**
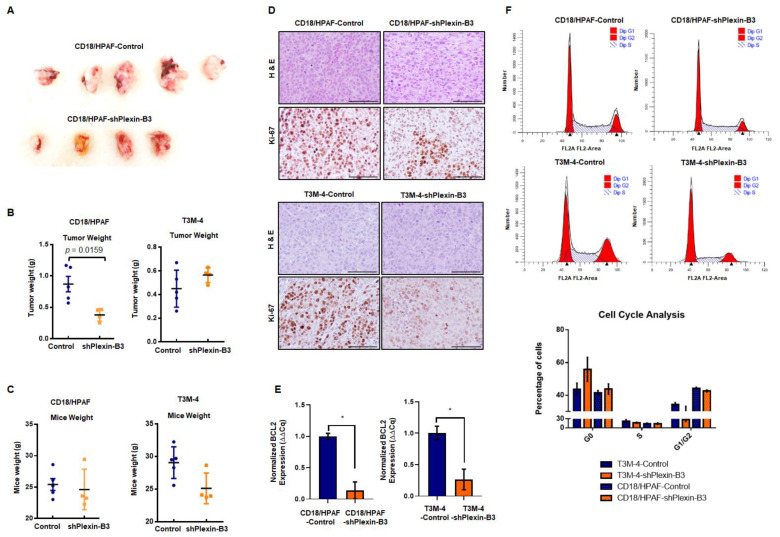
Loss of Plexin-B3 in PC cells results in a lower tumor burden in comparison with control cells. (**A**) Representative image of tumors formed from the injection of CD18/HPAF-Control and CD18/HPAF-shPlexin-B3 cells in athymic nude mice. The image shows the bigger tumor size of CD18/HPAF-Control cell-derived tumors in comparison with CD18/HPAF-shPlexin-B3-derived tumors. (**B**) Graph showing the average tumor weight of five different mice in each CD18/HPAF-Control and CD18/HPAF-shPlexin-B3 group and T3M-4-Control and T3M-4-shPlexin-B3 group. The graph shows a lower tumor burden of the CD18/HPAF-shPlexin B3 group in comparison with the CD18/HPAF-Control cells (*p* = 0.0162). (**C**) Graph showing the average mice weight of five different mice in each CD18/HPAF-Control and CD18/HPAF-shPlexin-B3 group and T3M-4-Control and T3M-4-shPlexin-B3 group. The graph shows no statistical difference in the mice weight of the T3M4- or CD18/HPAF-shPlexin-B3 cells-injected groups and their respective control cells-injected group. The graph’s values represent the tumor weight or mice weight, and error bars in the graphs represent the SEM. The statistical *p*-value was calculated using a nonparametric Mann–Whitney *U* test. (**D**) Representative images of Ki-67 IHC and H&E staining of the primary tumor burden formed by the CD18/HPAF-shPlexin-B3 or -control cells and T3M-4 -shPlexin-B3 or T3M-4 control cells showing lower Ki67-positive cells in the shPlexin-B3 group in comparison with their control cells group. The scale bar represents 100 μm. (**E**) Bar graph showing the relative fold decrease in the mRNA expression of *BCL2* in CD18/HPAF- and T3M4-shPlexin-B3 samples compared with their respective controls. *HPRT* is used as a control. The values are the mean relative fold changes ± SEM, bars (* *p* ≤ 0.05). (**F**) Flow cytometry showing a cell cycle analysis in CD18/HPAF-Control and CD18/HPAF-shPlexin-B3 cells and T3M-4-Control and T3M-4-shPlexin-B3 cells labeled with propidium iodide. The graph’s peaks correspond to the G1/G0 and G2/M phases of the cell cycle, while the dip represents the S-phase. The bar graph shows the percentages of unsynchronized CD18/HPAF or T3M-4-Control and CD18/HPAF or T3M-4-shPlexin-B3 in a different phase of the cell cycle. CD18/HPAF- or T3M-4-Control cells showed fewer cells in the S-phase than CD18/HPAF- or T3M-4-shPlexin-B3 cells.

CD18/HPAF-shPlexin-B3 or CD18/HPAF-Control cells were orthotopically injected into 4–6-week-old athymic female nude mice. We sacrificed the mice on the 34th day post-injection. We observed a significantly lower tumor weight (*p* = 0.0162) in mice injected with CD18/HPAF-shPlexin-B3 cells compared with mice bearing CD18/HPAF-Control cells (Figure 4A,B). However, we observed no significant difference in tumor weight in mice injected with T3M-4-shPlexin-B3 cells than in mice injected with T3M-4-Control cells (Figure 4B). Additionally, we did not observe any significant difference in the average weight of mice (*p* = 0.562) between both the groups (Figure 4C) of T3M-4 and CD18/HPAF control and shPlexin-B3 cells. 

To investigate the low tumor burden with the loss of Plexin-B3 at the molecular level, we performed an IHC of proliferation marker Ki-67 and observed a lower number of Ki-67-positive cells in tumors of mice injected with T3M-4- and CD18/HPAF-shPlexin-B3 in comparison with mice injected with T3M-4- and CD18/HPAF-Control cells (Figure 4D). Accordingly, the in vitro culture of T3M-4- and CD18/HPAF-shPlexin-B3 cells showed a lower relative transcription level of BCL2 in comparison with T3M-4- (*p* < 0.05) and CD18/HPAF-Control cells (*p* < 0.05) (Figure 4E). Lastly, the in vitro cell cycle analysis of CD18/HPAF- and T3M-4 control and their respective Plexin-B3 knockdown cells showed a higher number of G0 arrested cells in unsynchronized Plexin-B3 knockdown cells than unsynchronized control cells (Figure 4F).

### 2.5. Plexin-B3 Knockdown Enhanced Metastasis

**Figure 5:**
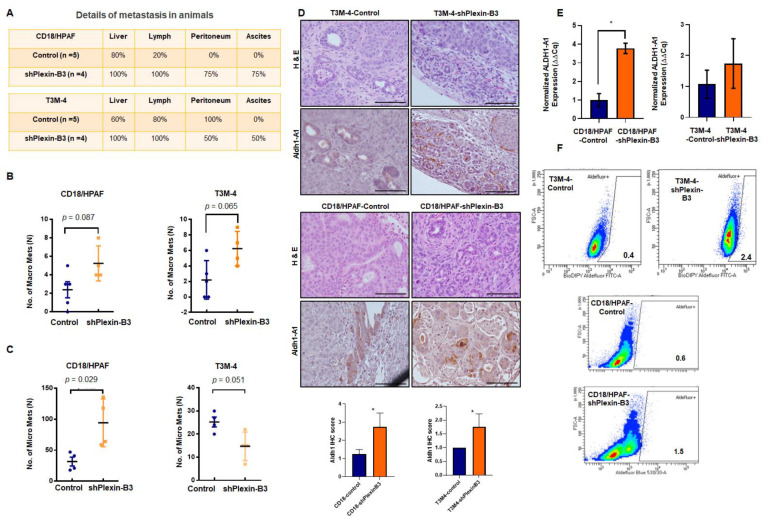
Loss of Plexin-B3 in PC cells enhances metastasis and induces stem cell-like characteristics compared to the control cells. (**A**) Chart of the percentage of metastasis incidence at different sites, like the liver, lymph node, peritoneum, and percentage of ascites formation in mice injected with CD18/HPAF- or T3M4- control and CD18/HPAF- or T3M-4-shPlexin-B3 cells. The chart shows a higher metastases and ascites incidence in mice injected with Plexin-B3 knockdown cells compared to the mice injected with control cells. (**B**,**C**) Graph showing the number of macro- (**B**) and micrometastases (**C**) in CD18/HPAF- or T3M-4-Control and CD18/HPAF- or T3M-4-shPlexin-B3 groups and the average number of metastases. (**B**) The graphs show a higher number of macrometastases formations in a group of mice injected with CD18/HPAF-shPlexin-B3 (*p* = 0.089) or T3M-4-shPlexin-B3 cells (*p* = 0.065) in comparison with the group injected with their respective control cells. (**C**) The graphs show a higher number of micrometastases formations in a group of mice injected with CD18/HPAF-shPlexin-B3 (*p* = 0.029) or T3M-4-shPlexin-B3 cells (*p* = 0.051) in comparison with the group injected with their respective control cells. The values in the graphs represent the number of macrometastases or micrometastases in the liver per mouse. The error bars in the graphs represent the SEM. The statistical *p*-value was calculated using a nonparametric Mann–Whitney *U* Test. (**D**) Representative images of ALDH1-A1 IHC and H&E staining of the primary tumor burden formed by the CD18/HPAF-shPlexin-B3 or -Control cells and T3M-4 -shPlexin-B3 or T3M-4 control cells showing higher ALDH1-A1-positive cells in the shPlexin-B3 group in comparison with their control cells group. The scale bar represents 100 μm. (* *p* ≤ 0.05) (**E**) Bar graph showing the relative fold increase in mRNA expression of *ALDH1-A1* in CD18/HPAF- and T3M4-shPlexin-B3 samples compared with their respective controls. *HPRT* is used as a control. The values are the mean relative fold changes ± SEM, bars. (* *p* ≤ 0.05) (**F**) Flow cytometry shows a higher ALDH1 expression using the ALDH1 enzymatic assay in T3M-4-shPlexin-B3 cells in vitro compared to T3M-4-Control cells. Diethylaminobenzaldehyde (DEAB), an inhibitor of ALDEFLUOR, was used as a negative control for each cell line.

We evaluated the incidence of metastasis in different organs, like the liver, peritoneal cavity, and lymph nodes and the number of micro- and macrometastases in the liver of mice injected with T3M-4 or CD18/HPAF-shPlexin-B3 and their respective control cells. We observed a higher incidence of liver, peritoneal cavity, and lymph node metastasis, along with the incidence of ascites in mice injected with Plexin-B3 knockdown cells compared to those injected with control cells (Figure 5A). Additionally, there were a high number of macrometastases in T3M-4-shPlexinB3 (*p* = 0.089) (Figure 5B) and CD18/HPAF-shPlexinB3 cells (*p* = 0.065) (Figure 5B), as well as high micrometastases in CD18/HPAF-shPlexinB3 cells (*p* = 0.029) (Figure 5C) in the liver of mice. However, we observed a lower number of micrometastases in the liver of mice injected with T3M-4-shPlexin-B3 cells than in mice injected with control cells (Figure 5C). The micrometastases in the liver formed by an injection of CD18/HPAF cells were bigger than those formed byT3M-4 cells (Figure S5). We also evaluated the epithelial–mesenchymal transition (EMT) markers in Plexin-B3 knockdown cells to gain mechanistic insight behind the loss of Plexin-B3-induced increased cellular motility. However, we observed no change in E-cadherin expression, N-cadherin, β-catenin, and snail at the protein levels (Figure S6) in CD18/HPAF T3M-4-shPlexin-B3 cells and their respective control cells. To investigate the high metastasis burden with the loss of Plexin-B3 at a molecular level, we performed an IHC of angiogenesis marker CD31 and stem cell markers ALDH1-A1 and CD44. We observed higher CD31 and CD44 staining of tumors formed by CD18/HPAF-shPlexin-B3 compared to the CD18/HPAF-Control cells, although a similar increase was absent in T3M-4-shPlexin-B3 as compared to the control tumors (Figure S7). We observed higher ALDH1-A1-positive cells in tumors formed by T3M-4- and CD18/HPAF-shPlexin-B3 cells than their respective control cells (Figure 5D). Accordingly, the in vitro culture of T3M-4- and CD18/HPAF-shPlexin-B3 cells showed a higher relative transcription level of ALDH-A1 in comparison with T3M-4- and CD18/HPAF-Control cells (*p* < 0.05) (Figure 5E). Lastly, we performed ALDH activity using an ALDEFLUOR enzymatic assay and observed a similar higher activity in T3M-4- and CD18/HPAF-shPlexin-B3 cells than T3M-4 and CD18/HPAF-Control cells (Figure 5F).

## 3. Discussion

In this study, we report the pathological expression and functional role of Plexin-B3 in PC and metastasis. Our group has previously delineated the role of Plexin-B3 ligand SEMA5A in PC tumors and metastases [13,14,19,20,21,22]. Our data demonstrated a disease stage-dependent Plexin-B3 expression, and the loss of Plexin-B3 enhanced the metastatic potential of the PC cells. Cancer cells, tumor stroma, and a normal human pancreas show a wide range of Plexin-B3 expressions. We also observed a higher Plexin-B3 IHC score in the tumors in comparison with a normal pancreas. Our Plexin-B3 IHC scores resonate with the Plexin-B3 expression in sequenced PC tumors evaluated through the online portal of MiPanda, where we observed significantly a higher Plexin-B3 expression (*p* = 1.51 × 10^−13^ in primary tumors in comparison with a normal pancreas. These observations were also supported by the gastric carcinoma patients’ samples demonstrating a higher expression of both receptor Plexin-B3 and its ligand SEMA5A in gastric carcinoma than the non-neoplastic tissue [23]. However, the IHC analysis of Plexin-B3 expression in the mouse pancreatic tumor progression model showed an initial increase of Plexin-B3 staining until 20 weeks, followed by a decrease in Plexin-B3 expression in the later stages with a positive Plexin-B3 expression in normal mice pancreas.

Similarly, results suggesting a loss of Plexin-B3 in PC were reported by Balakrishnan et al., showing somatic mutations in Plexin-B3 in PDAC cases [24]. Through functional assays in cellular models, the group demonstrated that C1613G, a R538H mutation in the extracellular domain of Plexin-B3, prevented the binding of the ligand Sema5A. These missense mutations resulted in the loss of Plexin-B3 function. Likewise, Liu et al. reported the downregulation of Plexin-B3 mRNA and protein expression in hepatocellular carcinoma samples compared to the corresponding adjacent noncancerous tissue [25]. Furthermore, this group elucidated the correlation between Plexin-B3 expression and clinicopathological data by performing IHC on 84 hepatocellular carcinoma archived specimens and showed a lower expression of Plexin-B3 in tumor tissues in comparison with the corresponding adjacent noncancerous tissues.

Using the tissue microarray containing matched primary pancreatic tumor cores with the corresponding liver metastatic sites cores, we observed a correlation of 0.56 between the Plexin-B3 expression in metastatic liver sites and their respective primary pancreatic tumors. Previously, our group showed a higher expression of plexin-B3 in the metastasis of prostate cancer in comparison to the primary tumor [14]. We also observed similar results for the expression of ligand SEMA5A in PC liver metastasis [21]. Similar to SEMA5A expression [21], Plexin-B3 expression was observed in the lymph node, peritoneum, and diaphragm metastases in KPC mice at the 50-week time point.

The knockdown of Plexin-B3 in two PC cell lines: -T3M-4 and -CD18/HPAF led to a marked difference in morphology compared to the control cells. The loss of Plexin-B3 increased the cellular spread of the cells with the remodeling of the actin filaments. We also observed the lack of three-dimensional colony formation in Plexin-B3 knockdown cells. Earlier, we have observed pronounced morphological changes associated with SEMA5A knockdown in these cell lines, leading to enhanced invasion and migration properties by the process of “epithelial–mesenchymal transition” or EMT [22]. Additionally, this increase in migration ability was associated with changes in the actin cytoskeleton’s polarized assembly. The actin cytoskeleton changes result in the formation of lamellipodia and filopodia that help the cell navigate through the matrix and the vasculature [26]. Contrary to the loss of SEMA5A associated with EMT, our present data demonstrated no changes in the different epithelial and mesenchymal markers with the loss of Plexin-B3.

We observed a higher in vitro cellular motility, migration, and invasion following the knockdown of Plexin-B3. Our in vitro functional assays explain the observed higher incidence of metastasis in different organs of mice injected with T3M4-/CD18/HPAF-Plexin-B3 knockdown cells in comparison with their respective -control cells. This increase in incidence and the number of metastasis was not a consequence of the primary tumor burden difference. Still, it was due to the increased invasiveness and migration properties of Plexin-B3 knockdown cells. Our results suggest that the loss of Plexin-B3 enhanced the escape of a tumor cell from the primary tumor by increasing the cancer cell’s motility and invasiveness, thereby resulting in a low tumor burden with high metastasis. Loss of the ligand SEMA5A also enhanced metastasis in PC but by undergoing EMT [22]. Similar to our observation, Plexin-B3 and ligand SEMA5A were found to inhibit human glioma cell invasion. Li et al. further showed that disrupting the SEMA5A/Plexin-B3 axis modulates the Rho family of GTPases, regulating cellular migration by reducing the actin cytoskeleton and disruption of vinculin-mediated focal attachments [27]. This group also demonstrated that the SEMA5A/Plexin-B3 axis causes the astrocytic differentiation of glioma cells [28]. Additionally, Plexin-B1 has been reported as a tumor suppressor in melanoma [29].

Our in vitro functional assays, such as the cell cycle analysis and low BCL2 expression in Plexin-B3 knockdown cells, explain our in vivo observation of a lower tumor burden with less Ki-67-positive cells in mice injected with Plexin-B3 knockdown cells. The loss of colony formation and defective proliferation appears to be the cause of a lower tumor burden. Our observations are supported by a recently published unrecognized function of the Semaphorin–Plexin-B2/B1 system as a crucial regulator of mitotic spindle orientation critical in establishing and maintaining the epithelial architecture during morphogenesis and repair [30].

Many recent reports have revealed that axon guidance cue molecules like the semaphorin/plexin axis are hijacked by different cancer cells to aid in their growth, differentiation, and renewal of cancer stem-like cells [31,32,33,34]. Our interest in investigating the role of Plexin-B3 in stem cell properties of PC cells came from various studies that demonstrate Plexin-B’s role in regulating the stemness of different cancers [30,35,36,37]. We examined two cancer stem cell markers: ALDH1-A1 and CD44 in the tumors and observed an increase in ALDH1-A1-positive cells in tumors compared to Plexin-B3 knockdown cells, suggesting that the loss of Plexin-B3 may directly or indirectly check cell proliferation and enhance cancer stemness in PC malignancy.

Our present study had an investigative approach towards exploring the possible role of Plexin-B3 in pancreatic cancer. In addition, semaphorins and their receptor plexins generally demonstrated dual functionality by either acting as tumor suppressors or tumor inducers in different cancers. We saw the tumor-specific and metastasis-specific roles of Semaphorin-5A, a ligand of Plexin-B3, in pancreatic cancer [21,22]. Additionally, plexins and semaphorins affect the stomal components, such as fibroblasts, endothelial cells, and immune cells, as well as tumor cells [38], thus increasing the possibility of dual effects on manipulating the expression of these molecules. Moreover, semaphorins and plexins are known players of immune infiltration and angiogenesis in tumor biology. Their roles are emerging in epithelial-to-mesenchymal plasticity and cancer stem cell characteristics [39], the major processes contributing to metastatic seeding and niches [40,41]. Thus, semaphorins/plexins can orchestrate different aspects of metastatic colonization and metastatic niches. 

However, our current study has certain limitations: we used a selected subpopulation of the cells with Plexin-B3 knockdown. Further experimentation using an inducible expression system and better cellular model systems is needed. Furthermore, our study lacks a definitive mechanistic explanation of the suggested functional role of Plexin-B3 and limits our observations more to associations and possibly indirect phenomena rather than causative effects. In the future, SEMA5A-Plexin-B3-specific signal transduction and cell signaling, particularly in relation to the cancer stemness feature and whether the loss of Plexin-B3 is mechanistically required for the extra-pancreatic spread, needs evaluation.

## 4. Materials and Methods

### 4.1. Human PC Patient Samples

We obtained tissue microarray (TMA) slides from the Rapid Autopsy Program of the University of Nebraska Medical Center Omaha (UNMC, NE, USA). TMA 2013 #1-1 contained cores of twelve PC tumor cases, two nonmatched normal pancreases, ten metastatic liver, and one normal liver. TMA 2012 #2-1 and TMA 2012 #3-1 contained matched cores of the metastatic liver (TMA 2012 #2-1) and the corresponding PC tumors (TMA 2012 #3-1) from twenty-five patients. Table 1 contains the details of the normal and pathological specimens of the pancreas and liver in different TMA.

### 4.2. Mouse Model of PC Disease Progression and Metastasis Specimens

The groups of David Tuveson and Ronald DePinho utilized the K-ras^LSL.G12D^ mouse to generate a new model of PDAC (KC model) [42]. All pancreatic cells were derived from pancreatic progenitors with transcription factor PDX. This model showed that the K-ras mutation was sufficient to initiate pancreatic cancer formation in mice and recapitulated the stepwise development of cancer seen in humans. However, this model had a long latency period and infrequent progression to invasive carcinoma. On the other hand, the KPC mice had a p53^R172H^ conditional expression that accelerated K-ras^G12D^ pancreatic tumorigenesis [42,43]. The KPC model developed PanIN lesions at an accelerated rate, with a median survival of the mice of 5.5 months. Nearly 80% of the animals showed metastases (liver, lung, and peritoneum) at the same sites as seen in human PDAC patients.

We utilized tissue sections of primary pancreatic tumors of the KC mouse model obtained from different time points/ages (10, 20, 30, and 50 weeks) and different metastatic sites of KPC mice at the 50-week time point, such as the liver, lymph node, peritoneum muscle, and diaphragm muscle.

### 4.3. Pancreatic Ductal Adenocarcinoma (PDAC) Database

We explored the online portal MiPanda to find Plexin-B3 expression in the normal pancreas and the pancreatic tumor samples. The MiPanda portal contained next-generation sequencing data for 419 pancreatic cancer cases [44]. We also utilized the GEPIA portal for a survival analysis of pancreatic ductal adenocarcinoma patients with respect to Plexin-B3 expression [45].

### 4.4. Cell Lines and Culture Conditions

We selected the T3M-4, and CD18/HPAF cell lines, as both T3M-4 and CD18/HPAF cell lines are adherent in nature, derived from the metastatic site of PDAC patients, and form well-differentiated tumors in athymic mice. We screened pancreatic cancer cell lines for the expression analysis of receptor Plexin-B3 and its ligand SEMA-5A [19]. Both T3M-4 and CD18/HPAF cell lines showed a high endogenous expression of Plexin-B3 and SEMA5A, the criteria we chose for the selection of cell lines used for the generation of knockdown of Plexin-B3, SEMA5A, and double-knockdown if needed. 

T3M-4 and CD18/HPAF cell lines were grown in Dulbecco’s Modified Eagle’s Medium (Sigma-Aldrich, St. Louis, MO, USA) supplemented with 5% fetal bovine serum (FBS) (Sigma-Aldrich), 2-mM L-glutamine (Mediatech, Herdon, VA, USA), 1% vitamins (Mediatech), and 0.08% gentamycin (Invitrogen, Carlsbad, CA, USA). We tested the cell lines for mycoplasma using a MycoAlert Plus Mycoplasma Detection kit (Lonza, Rockland, ME, USA). Human DNA Identification Laboratory, UNMC, Omaha, NE, USA authenticated the T3M-4 and CD18/HPAF cell lines by performing short tandem repeat (STR) tests. The group evaluated fifteen STR markers (D8S1179, D21S11, D7S820, CSF1PO, D3S1358, THO1, D13S317, D16S539, D2S1338, D19S433, vWA, TPOX, D18S51, D5S818, and FGA) and the gender marker Amelogenin.

### 4.5. Generation of Plexin-B3 Knockdown Cell Lines

We described the information of the vector pSuper.neo containing specific oligos of Plexin-B3-specific oligo and scramble in our previous study [20]. We also previously described the generation of Plexin-B3 knockdown in T3M-4 and CD18/HPAF cell lines [21]. In brief, we selected and maintained a mixed population of stably transfected cells using G418 Disulphate salt (Sigma-Aldrich). We maintained T3M-4- and CD18/HPAF control/shPlexin-B3 cell lines using 1000 µg/mL and 600 µg/mL of G418 Disulphate salt. 

### 4.6. mRNA Analysis

We plated T3M-4- and CD18/HPAF-shPlexin-B3 and their respective control cells at 6 × 10^6^ in a 100-mm dish for mRNA analysis. We performed RNA isolation and cDNA preparation as described previously [22]. We prepared quantitative Real Time-Polymerase Chain Reactions (qRT-PCR) using PowerUp™ SYBR™ Green Master Mix (Thermo Fisher, Carlsbad, CA, USA), complementary DNA, gene-specific primers, and nuclease-free water. The results were analyzed using Thermo Fisher Connect (Thermo Fisher). Mean C_t_ values of the target genes were normalized to mean C_t_ values of the endogenous control, hypoxanthine-guanine phosphoribosyltransferase (HPRT) (−∆C_t_ = C_t_ (HPRT) − C_t_ (target gene)). We calculated the mRNA expression of target genes versus HPRT (2^(−∆Ct)^), analyzed the melting curve, and used 1% agarose gels to resolve the amplified product. The details of the primers are PLXNB3- Fwd (forward): AGGGCGAGAGGACCATCTAC, Rev (reverse): GCCTCGGAAATGTTGAAGGTT, BCL-2- Fwd: TCCATGTCTTTGGACAACCA, Rev: CTC CACCAGTGTTCCCATCT, ALDH1-A1- Fwd: CCATAACAATCTCCTCTGCTC, Rev: CTCCCAGTTCTCTTCCATTTC, HPRT- Fwd: AGGGTGTTTATTCCTCATGGAC, and Rev: GTAATCCAGCAGGTCAGCAAAG.

### 4.7. Immunoblotting

We seeded 1 × 10^6^ cells per well on a 6-well plate for protein isolation. We washed the cells three times using Phosphate Buffer Saline and prepared the lysate using Membrane Lysis Buffer (M-PER^®^, Pierce, Rockford, IL, USA) containing protease inhibitors (Complete Mini, Roche Diagnostics, Mannheim, Germany). We incubated the cell lysate on ice for 30 min, followed by centrifugation at 10,000 rpm for 10 min at 4 °C. Following incubation, we transferred the supernatant into a fresh tube and performed the protein quantitation using a BCA kit (Pierce^™^ BCA Protein Assay Kit (Thermo Fisher Scientific, Rockford, IL, USA). Quantified protein (25 μg) was denatured using the SDS Laemmli buffer [46]. We used 10% SDS-PAGE at a constant voltage of 100 V to separate the denatured proteins until the dye reached the end of the gel and transferred the separated proteins to a 0.45-μm Polyvinylidene fluoride (PVDF) membrane (Millipore, Billerica, MA, USA) at a constant voltage of 100 V for 90 min. Next, we blocked the membrane with 3% BSA in 0.1% Tween containing Tris Buffer Saline (TBST) for an hour at room temperature and incubated the membrane with a respective primary antibody at 4 °C overnight. We used the following primary antibodies for our study: Plexin-B3 (Santa Cruz, Dallas, TX, USA sc671441, 1:200), αE-cadherin (HECD1; 1:100), αN-cadherin (1:100), αβ-catenin (1:200) (E-cadherin, N-cadherin, and β-catenin were a kind gift from Dr. Keith Johnson’s lab, UNMC), SNAIL (Abcam, Cambridge MA, ab53519; 1:500), αGAPDH (Santa Cruz- sc-59541; 1:1000), and αβ-actin (Sigma-2066, 1:1000). The next day, we washed the membrane with TBST for 10 min three times. After washing, the blots were incubated with secondary horseradish peroxidase antibodies (mouse (Sigma, ab97023, 1:5000) and rabbit (Thermo Fisher Scientific, 31460, 1:5000) for an hour at room temperature. The membrane was washed with TBST for 10 min three times. Finally, we developed the blots using the Luminata^TM^ Forte (Millipore, Billerica, MA, USA) on the Molecular Imager^®^ Gel Doc™ XR System (BIO-RAD, Hercules, CA, USA) using Image Lab version 5.2.1. The intensity of the bands obtained from immunoblotting was measured using ImageJ software (National Institute of Health, Bethesda, MD, USA). We quantified the peaks for our protein of interest and their respective loading control and normalized the bands to the control cells used in the study.

### 4.8. Immunofluorescence

We plated 1 × 10^5^ cells on 22 × 22-mm coverslips (Fisher, Pittsburgh, PA, USA), placed them in 6-well plates to adhere overnight, and performed immunofluorescence as described previously [22]. In brief, we stained the actin cytoskeleton using Texas Red Phalloidin (Molecular Probes, Eugene, OR, USA) for 20 min and observed immunofluorescence using a Nikon fluorescent microscope (Melville, NY, USA) with NIS Element software.

### 4.9. Three-Dimensional Colony Formation Assay

We thawed the growth factor for the three-dimensional colony formation assay-reduced Matrigel (Corning, Corning, NY, USA) on ice. Cells (1 × 10^4^ cells per mL) suspended in serum-free media were diluted with Matrigel in a 1:1 ratio. Next, we took the 96-well plate and kept it on ice. We added 150 μL of the prepared cells suspension per well of a chilled 96-well plate. The 96-well plate was incubated at 37 °C in a humidified 5% CO_2_ atmosphere for half an hour. After incubation, we added 150-μL media per well and left the plate undisturbed for 72 h. Colonies were visualized after 72 h using a Life EVOS fluorescence microscope (Life Technologies, Grand Island, NY, USA).

### 4.10. Wound-Healing Assay

We plated 0.2 × 10^6^ cells per well in 12-well plates and allowed them to reach 90–95% confluency before generating a wound using a 1-mL pipette tip (Thermo Fisher Scientific). We washed the cells with HBSS (Hank’s Balanced Salt Solution, Sigma-Aldrich, St. Louis, MO, USA) and incubated them with a medium containing 5% serum for 24 h. Cells were photographed under an inverted microscope at 40× magnification at times T = 0 h and T = 24 h. The width of the wound was measured using ImageJ software. Distance migrated was calculated by the formula: initial wound width (T = 0 h) − final wound width (T = 24 h)/(T = 0 h) × 100.

### 4.11. Invasion Assay

We performed an invasion assay using Transwell Corning® BioCoat™ Matrigel® Invasion Chambers with 8.0-μm polyester (PET) membrane in 6-well Plates (Corning Costar Corp., Cambridge, MA, USA). Cells (1 × 10^5^) were plated onto Transwell chambers with serum-containing media and incubated at 37 °C in 5% CO_2_ for 24 h. After 24 h, we removed the cells from the top of the Transwell chambers using a cotton swab (residual cells). We stained the membrane using a Hema 3 kit (Fisher Scientific Company LLC, Kalamazoo, MI, USA), as per the manufacturer’s instructions. Cells were counted in five independent high-powered fields (200×) using a Nikon microscope. 

### 4.12. Migration Assay

We performed the migration assay using Transwell chambers with a polycarbonate membrane containing 8.0-µm pores (Corning Costar Corp.). We plated cells (1 × 10^5^) onto Transwell chambers with serum-free media, and the setup was incubated at 37 °C in 5% CO_2_ for 24 h. The remaining protocol was similar to the one described above for invasion assay. 

### 4.13. Xenogenic Mouse Models

We maintained the mice under specific pathogen-free conditions and performed all procedures using the institutional guidelines and were approved by the UNMC’s Institutional Animal Care and Use Committee (IACUC, Omaha, NE, USA). We purchased the female athymic BALB/c nude mice (NCI-nu, 6–8-week-old) from the National Cancer Institute (Bethesda, MD, USA). For the orthotopic injection per animal, we used 2.5 × 10^5^ cells in 50 μL of T3M-4-Control, T3M-4-shPlexin-B3, CD18/HPAF-Control, and CD18/HPAF-shPlexin-B3. We performed cell preparation and followed the protocol as described by Sadanandam et al. [19]. Wound clips were removed at approximately 10–14 days post-surgery. Mice were monitored for tumor growth and sacrificed 34 days post-injection. Primary tumors and metastases were resected and processed for further analysis.

### 4.14. IHC

We performed IHC as described previously by Awaji et al. [47]. In brief, we incubated slides with primary antibodies, such as αPlexin-B3 (Santa Cruz-sc671441; 1:100), αKi67 (1:100; Santa Cruz-sc15402), αALDH1-A1(1:100; sc374149), αCD31 (1:50, Abcam,-ab28364), and αCD44 (1:500; Abcam-ab157107), in antibody diluent overnight at 4 °C. The next morning, we washed the slides and incubated with biotinylated anti-rabbit or anti-mouse secondary antibody (Vector Laboratories, Burlingame, CA, USA) for 45 min. Images were captured through the Nikon microscope using NIS Element software.

IHC scoring was performed according to the following criteria: percentage of positive cells on the slides was as follows: 0 (negative), 0.1 (1–10% of cells positive), 0.2 (11–20% of cells positive), and 0.3 (20–30% of cells positive), and so forth. Furthermore, the intensity was designated as weak (1 point), moderate (2 points), strong (3 points), or very strong (4). The IHC composite score was calculated by multiplying the extent of positive cells with intensity. Two independent observers examined each slide, and their observations were positively correlated with each other. Average scores were used for analysis, and if the two observers significantly differed in their scoring, a third observer examined the slide.

### 4.15. Propidium Iodide Staining

We seeded 1 × 10^6^ cells in a 6-well plate overnight for cell cycle analysis. The propidium iodide (Roche, Indianapolis, IN, USA) staining was performed as described previously [22]. Data were acquired using a FACSCalibur^TM^ flow cytometer (BD Biosciences, Franklin Lakes, NJ, USA).

### 4.16. Aldefluor Assay

We seeded 1 × 10^6^ cells in a 6-well plate overnight for Aldefluor analysis. Aldefluor levels in T3M-4- and CD18/HPAF-shPlexin-B3 and their respective controls cells were determined using an ALDEFLUOR^TM^ assay kit (Stem Cell Technologies Waltham, MA, USA). As described previously [48], we performed the Aldefluor assay and evaluated the results as per the manufacturer’s protocols. 

### 4.17. Statistical Analysis

We performed the statistical analysis using GraphPad Prism software (GraphPad Software version 9, Inc., La Jolla, CA, USA). We used either a Student’s *t*-test or nonparametric Mann–Whitney *U* test or a two-way ANOVA test for multiple comparison groups for data evaluation and comparisons. A *p*-value of less than 0.05 was considered significant. The normal distribution of the GEO dataset GDS4103 using a box plot analysis was analyzed using SPSS software (SPSS, Inc., Chicago, IL, USA). 

## 5. Conclusions

**Figure 6:**
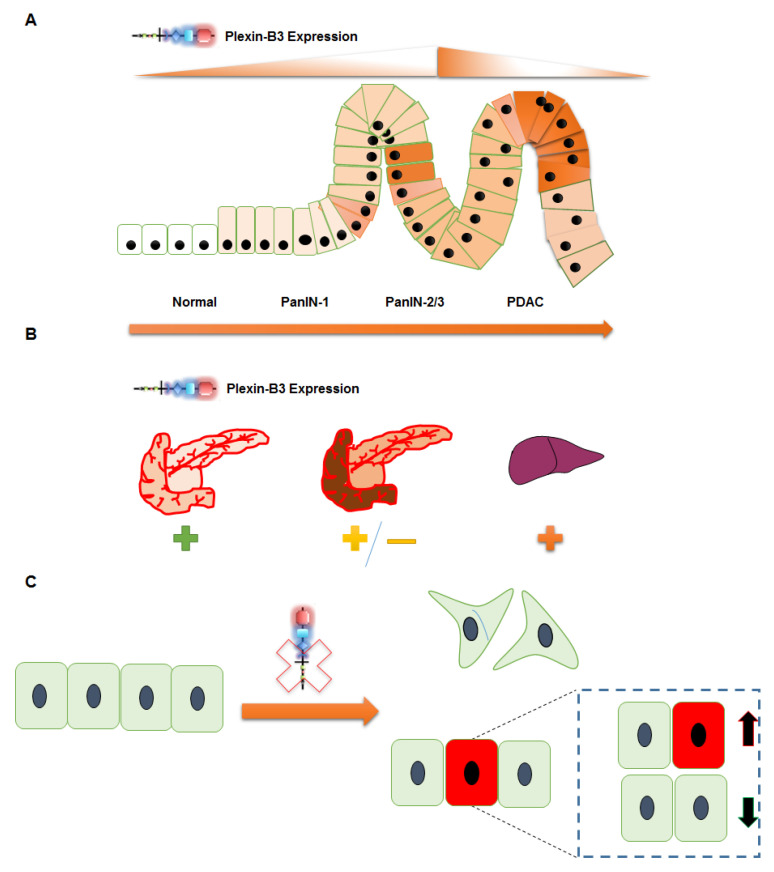
Schematic representation summarizing the role of Plexin-B3 in PC. (**A**–**C**) Plexin-B3 expression analysis in different PC mouse models and human patient samples in a tissue microarray (TMA) suggests (**A**) an initial increase followed by a decrease in Plexin-B3 expression with PC progression. (**B**) Normal pancreas and different PC metastatic sites, like the liver, diaphragm muscles, and others, are positive for the expression of Plexin-B3. The expression of Plexin-B3 is stage-dependent in PC. (**C**) The loss of Plexin-B3 remodels the PC cellular shape, actin filaments, and enhances the invasiveness and migration abilities that facilitate metastasis. The loss of Plexin-B3 can directly or indirectly check cell division (represented by green color cells) and enhances stem cell-like PC cells (represented by red color cells).

In conclusion, we report novel insights into the pathological expression and functional role of Plexin-B3 in PC tumor growth and metastasis (Figure 6). The loss of Plexin-B3 accelerates metastasis in PC by enhancing the migration, invasiveness, and altering these cells’ actin cytoskeleton. Furthermore, our data suggest that the loss of Plexin-B3 directly or indirectly interferes with cell division or proliferation and induces cancer stem cell-like PC cells.

## Figures and Tables

**Table 1:** Number and type of specimen in different TMA utilized in the study.

S. No.	TMA Identity	Normal Pancreas	Tumor	Normal Liver	Metastatic Liver
1	TMA 2013 #1-1	2	12	2	10
2	TMA 2012 #2-1	1	0	1	25
3	TMA 2012 #3-1	2	25	0	0

## Data Availability

The data presented in this study are available in the article or supplementary material published.

## Supplementary Materials

The following are available online at https://www.mdpi.com/2072-6694/13/4/818/s1, Figure S1: High and low Plexin-B3 expression results in no difference in the Overall survival and Disease-free survival of PC patients, Figure S2: Knockdown of Plexin-B3 in T3M-4 and CD18/HPAF cells, Figure S3: Loss of Plexin-B3 results in no difference in the compact colony formation ability of T3M-4-Control and T3M-4-shPlexin-B3 cells, Figure S4: Loss of Plexin-B3 results in no difference in the invasiveness of T3M-4-Control and T3M-4-shPlexin-B3 cells, Figure S5: Metastasis in the liver of mice injected with the Plexin-B3 knock-down cells in comparison with the Control cells, Figure S6: Loss of Plexin-B3 shows no difference in the expression of Epithelial and Mesenchymal markers, Figure S7: Angiogenesis (CD31) and stem cell marker (CD44) expression in tumors formed by Plexin-B3 knock-down cells and their respective Control cells.
