# Supplementary material for: Plexin-B3 Regulates Cellular Motility, Invasiveness, and Metastasis in Pancreatic Cancer
Source: Cancers (Basel). 2021 Feb 16;13(4):818. doi: 10.3390/cancers13040818 (PMC7919786; doi:10.3390/cancers13040818)
Supplement: Supplementary file 1 [file cancers-13-00818-s001.pdf]

Plexin-B3 Regulates Cellular Motility, Invasiveness, and Metastasis in Pancreatic Cancer

Sugandha Saxena, Dipakkumar R. Prajapati, Paran Goel, Babita Tomar, Yuri Hayashi, Pranita Atri, Satyanarayana Rachagani, Paul M. Grandgenett, Michael A. Hollingsworth, Surinder K. Batra and Rakesh K. Singh

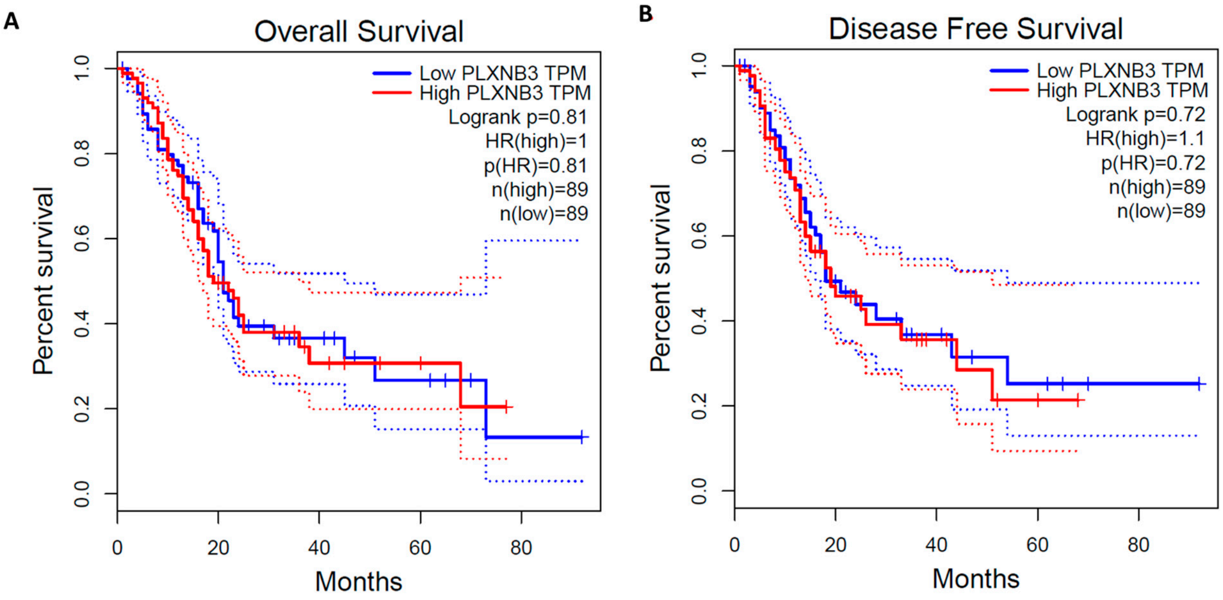

Figure S1. High and low Plexin-B3 expression results in no difference in the Overall survival and Disease-free survival of PC patients. Graph showing no statistical difference in the (A) Overall survival and (B) Disease-free survival with high and low cut off of Plexin-B3 expression in PC patients. The analysis is derived from the GEPIA portal.

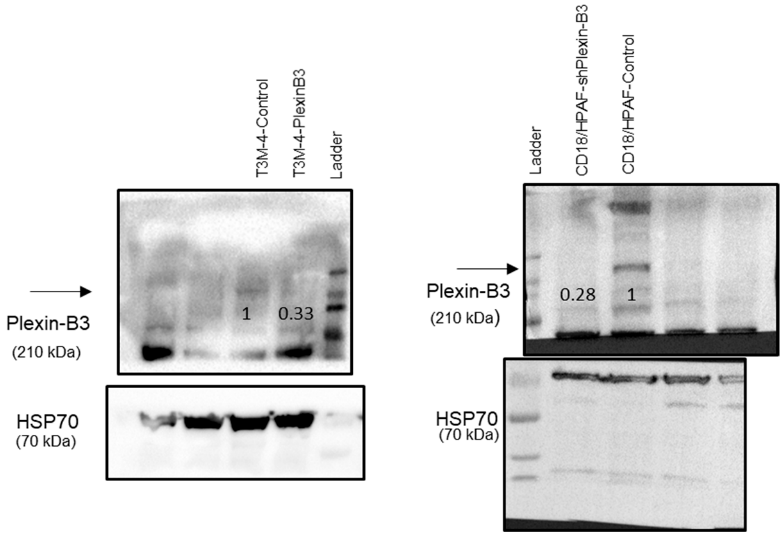

Figure S2. Knockdown of Plexin-B3 in T3M-4 and CD18/HPAF cells Western blot analysis of whole-cell lysates of CD18/HPAF-and T3M-4-control and -Plexin B3 knockdown cells. The Plexin-B3 expression is downregulated in Plexin-B3 knockdown cells of T3M-4- and CD18/HPAF compared to their respective control cells.

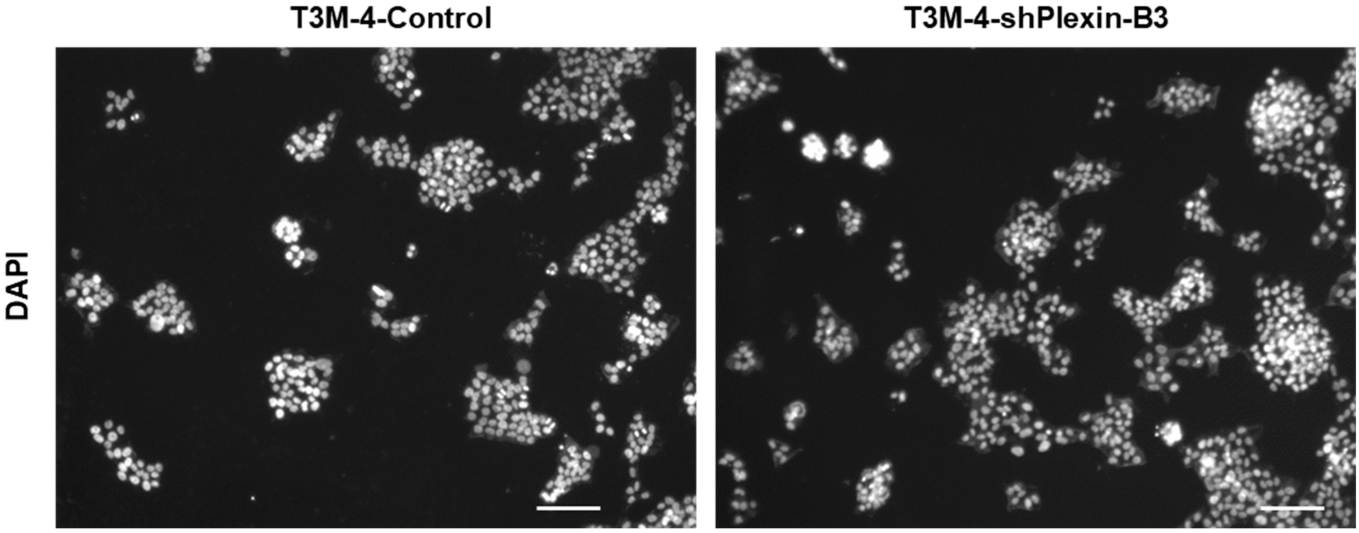

Figure S3. Loss of Plexin-B3 results in no difference in the compact colony formation ability of T3M-4-Control and T3M-4-shPlexin-B3 cells. Representative pictures showing no morphological difference in the compact colony formation ability of T3M-4-Control and T3M-4-shPlexin-B3 cells grown of three-dimensional culture using matrigel matrix. Scale bar represents 100 µm.

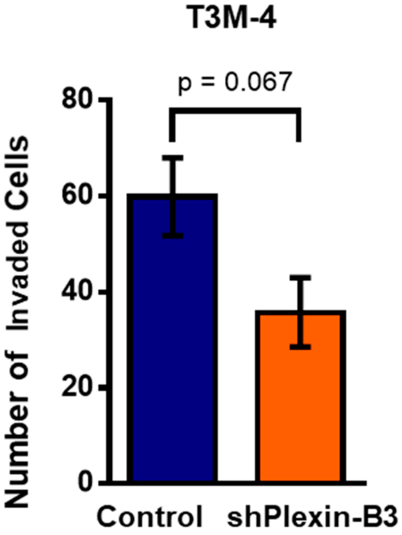

Figure S4. Loss of Plexin-B3 results in no difference in the invasiveness of T3M-4-Control and T3M-4-shPlexin-B3 cells**.** Bar graph showing no statistical difference in the number of invaded cells in T3M-4-Control and T3M-4 Plexin-B3 knockdown cells (p = 0.067). The graph's values represent the number of invaded cells, and the error bars represent Standard Error of Mean. Statistical p-value was calculated using a non-parametric Mann-Whitney U-Test.

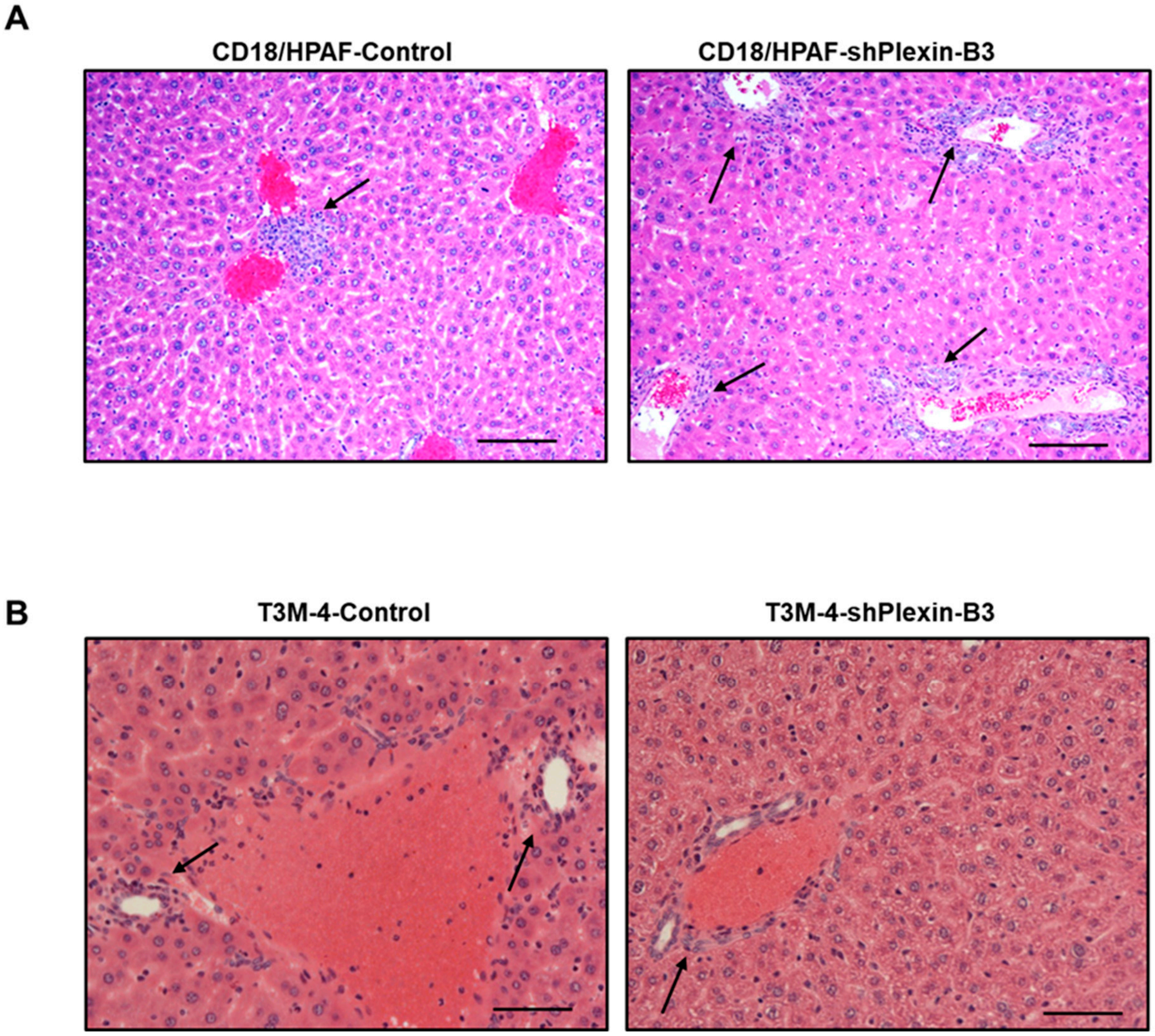

Figure S5. Metastasis in the liver of mice injected with the Plexin-B3 knock-down cells in comparison with the Control cells. (A) Representative images of H & E staining of the liver showing a higher number of micrometastases in the liver in a group of mice injected with CD18/HPAF-shPlexin-B3 cells in comparison with the group injected with CD18/HPAF-Control cells (B) Representative images of H & E staining of the liver showing micrometastases in the liver in a group of mice injected with T3M-4-shPlexin-B3 cells in comparison with the group injected with T3M-4-Control cells.

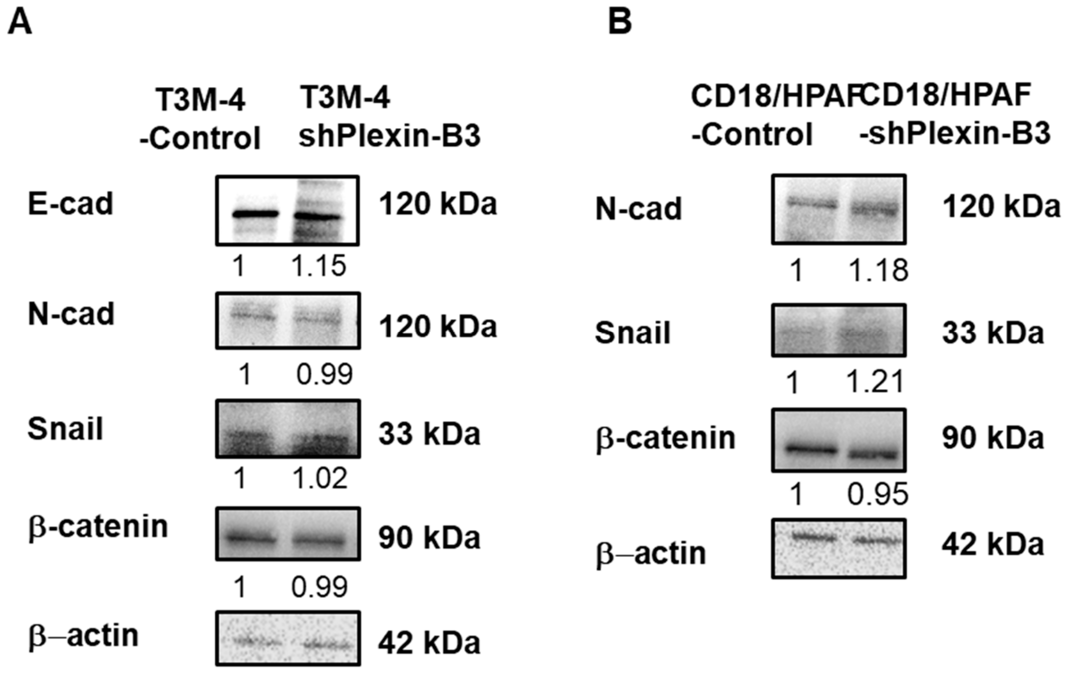
A
T3M-4
-Control
T3M-4
shPlexin-B3
E-cad
120 kDa
1 1.15
N-cad
120 kDa
1 0.99
Snail
33 kDa
1 1.02
β-catenin
90 kDa
1 0.99
β–actin
42 kDa
B
CD18/HPAF
-Control
CD18/HPAF
-shPlexin-B3
N-cad
120 kDa
1 1.18
Snail
33 kDa
1 1.21
β-catenin
90 kDa
1 0.95
β–actin
42 kDa

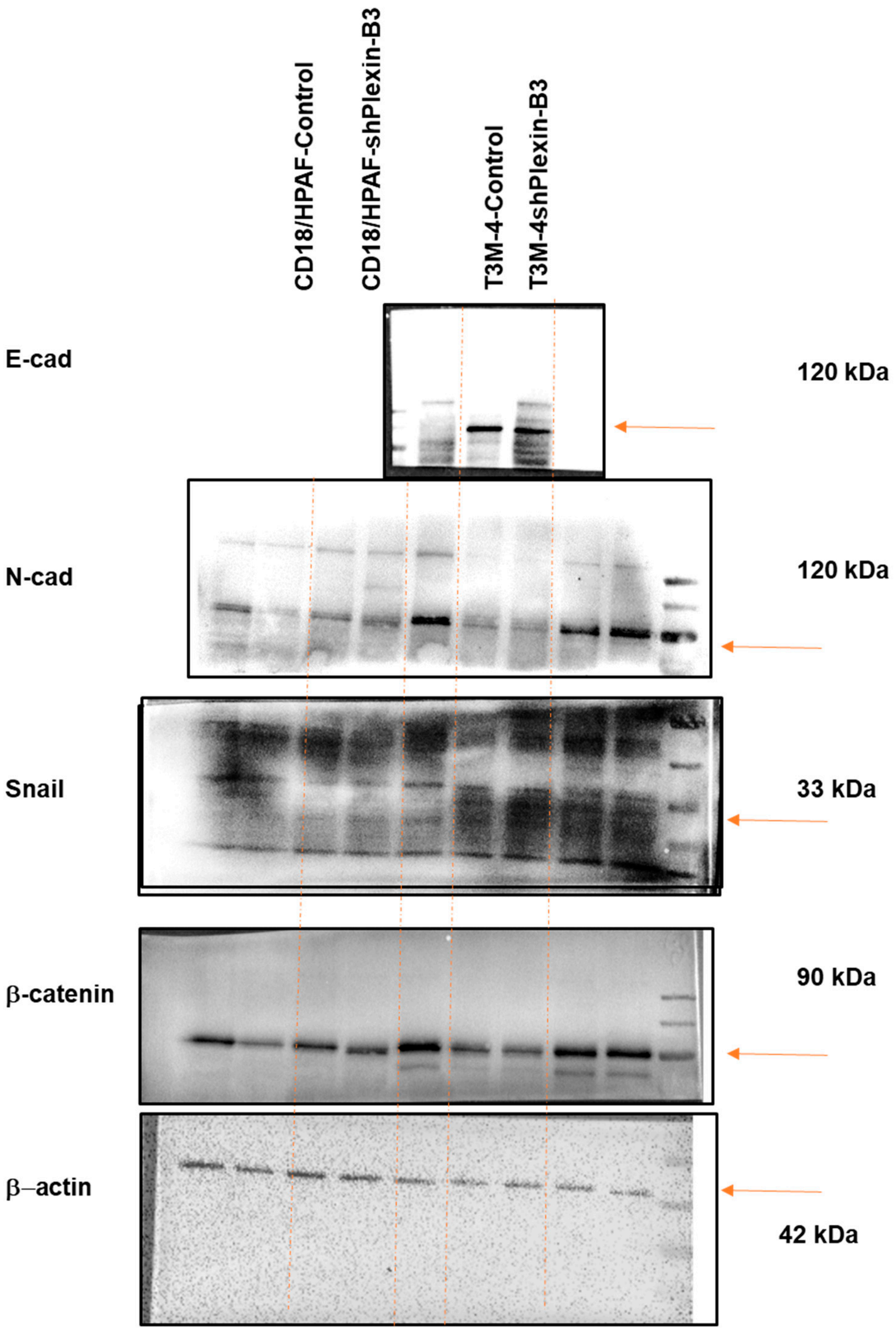

Figure S6. Loss of Plexin-B3 shows no difference in the expression of Epithelial and Mesenchymal markers**. (A,B)** Western blot analysis of whole-cell lysates of T3M-4 and CD18/HPAF Control and Plexin-B3 knock-down cell lysates. Western blot analysis shows no change in E-cadherin expression, N-cadherin, β-catenin, and Snail at protein levels in T3M-4- (A) CD18/HPAF shPlexin-B3 cells (B) and their respective Control cells. The intensity of the bands in western blot analysis was quantified by Software Image J using β-actin as an internal control, and it was normalized with the T3M-4 or CD18/HPAF control cells.

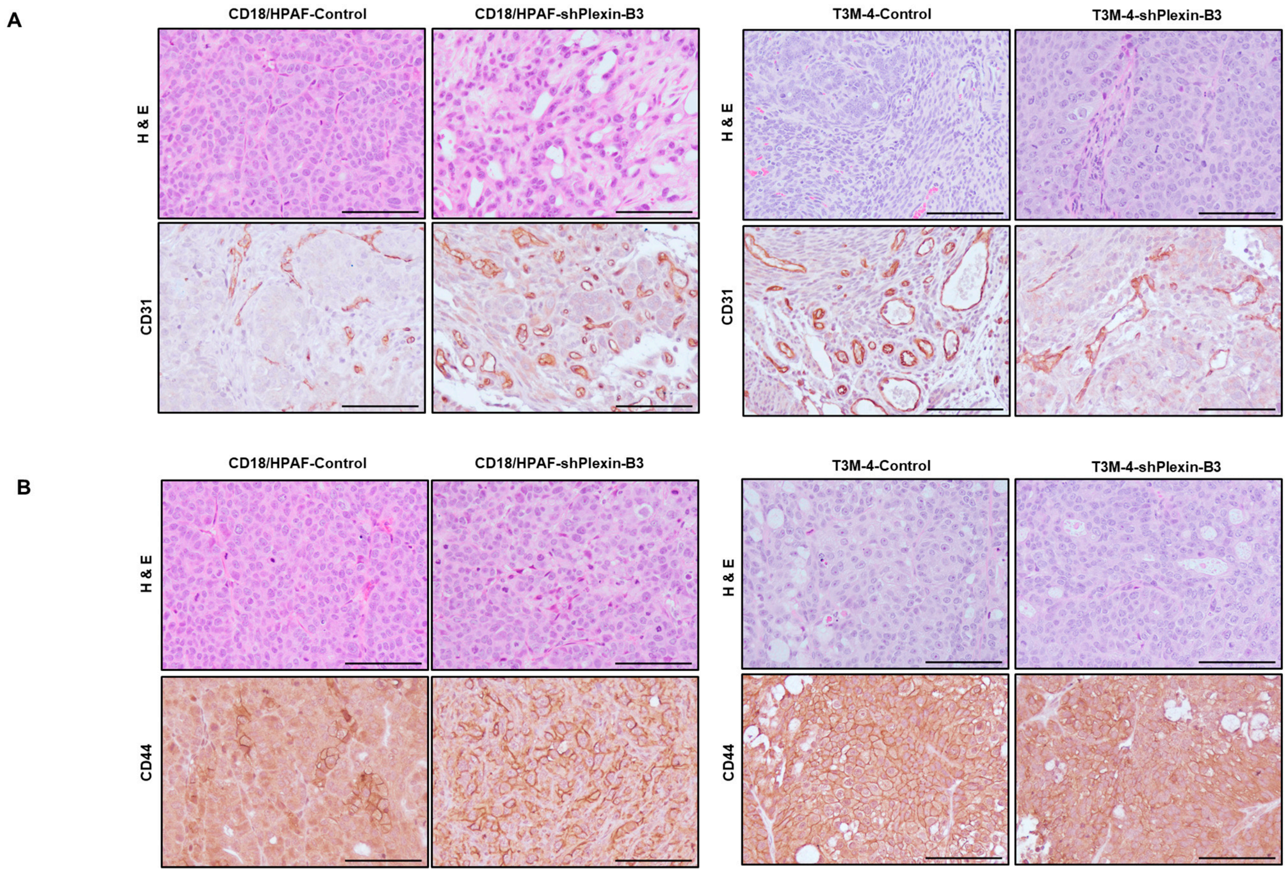

Figure S7. Angiogenesis (CD31) and stem cell marker (CD44) expression in tumors formed by Plexin-B3 knock-down cells and their respective Control cells. (A) Representative images of angiogenesis marker CD31 IHC and H and E staining of primary tumor burden formed by the CD18/HPAF-shPlexin-B3 or-Control cells and T3M-4 -shPlexin-B3 or T3M-4 Control cells. The images show an increase in CD31 staining of CD18/HPAF-shPlexin-B3 tumors; however, there was no similar increase in T3M-4 -shPlexin-B3 compared to their respective control cells. (B) Representative images of stem cell marker CD44 IHC and H and E staining of primary tumor burden formed by the CD18/HPAF-shPlexin-B3 or-Control cells and T3M-4 -shPlexin-B3 or T3M-4 Control cells. The images show an increase in CD44 staining of CD18/HPAF-shPlexin-B3 tumors; however, there was no similar increase in T3M-4 -shPlexin-B3 compared to their respective control cells. The scale bar represents 100 µm.
